# Genome Sequence of *Methanosarcina soligelidi* SMA-21, Isolated from Siberian Permafrost-Affected Soil

**DOI:** 10.1128/genomeA.00270-15

**Published:** 2015-04-23

**Authors:** Mashal Alawi, Nicole Shapiro, Tanja Woyke, Fabian Horn, Corien Bakermans, Dirk Wagner

**Affiliations:** a GFZ German Research Centre for Geosciences, Section 4.5 Geomicrobiology, Potsdam, Germany; b DOE Joint Genome Institute, Walnut Creek, California, USA; c Altoona College, The Pennsylvania State University, Altoona, Pennsylvania, USA

## Abstract

Here, we announce the genome sequence of *Methanosarcina soligelidi* SMA-21, an anaerobic methanogenic archaeon that was previously isolated from Siberian permafrost-affected soil. The sequencing of strain SMA-21 yielded a 4.06-Mb genome with 41.5% G+C content, containing a total of 2,647 open reading frames.

## GENOME ANNOUNCEMENT

*Methanosarcina soligelidi* (type strain SMA-21 = DSM 26065 = JCM 18468) was initially isolated from Siberian permafrost-affected soil and described as a novel species within the order *Methanosarcinales* (1). The 16S rRNA gene sequence (NCBI AB973359) was close to that of *Methanosarcina mazei* (99.9%). The strain grows on H_2_/CO_2_, methanol, and acetate and has a high survival potential against air exposure, desiccation, freeze-thaw cycles, and long-term freezing.

Here, we report the complete genome sequence of *M. soligelidi* SMA-21. The genome was generated at the Department of Energy (DOE) Joint Genome Institute (JGI) using the Pacific Biosciences (PacBio) sequencing technology (2). A PacBio SMRTbell library was constructed and sequenced on the PacBio RS platform, which generated 164,129 filtered subreads totaling 551.7 Mbp. The raw reads were assembled using HGAP (version 2.1.1) (3). The final draft assembly contained 1 contig in 1 scaffold, totaling 4.1 Mbp in size (G+C content, 41.50%). Genes were identified using Prodigal (4), followed by a round of manual curation using GenePRIMP (5). The tRNAscan-SE tool (6) was used to find tRNA genes, whereas rRNA genes were found by searches against models of the rRNA genes built from SILVA (7). Other noncoding RNAs, such as the RNA components of the protein secretion complex and RNase P, were identified by searching the genome for the corresponding Rfam profiles using Infernal (http://infernal.janelia.org). Additional gene prediction analysis and manual functional annotation were performed within the Integrated Microbial Genomes (IMG) platform (8) developed by JGI, Walnut Creek, CA, USA (http://img.jgi.doe.gov). The genome contains 3,440 protein-coding sequences, of which 75.4% (2,647) had a predicted function. Likewise, the genome contains five clustered regularly interspaced short palindrome repeat (CRISPR) loci and CRISPR-associated proteins (Cas). The genetic, metabolic, and physiological features of the species will be unveiled by future comparative genomic analyses.

### Nucleotide sequence accession number. 

This whole-genome shotgun project has been deposited in DDBJ/EMBL/GenBank under the accession no. JQLR00000000. The version described in this paper is the first version.
